# Laminin-111 peptide C16 regulates invadopodia activity of malignant cells through β1 integrin, Src and ERK 1/2

**DOI:** 10.18632/oncotarget.10062

**Published:** 2016-06-15

**Authors:** Adriane S. Siqueira, Monique P. Pinto, Mário C. Cruz, Basilio Smuczek, Karen S.P. Cruz, José Alexandre M. Barbuto, Daisuke Hoshino, Alissa M. Weaver, Vanessa M. Freitas, Ruy G. Jaeger

**Affiliations:** ^1^ Department of Cell and Developmental Biology, Institute of Biomedical Sciences, University of Sao Paulo, Sao Paulo, SP, 05508-000, Brazil; ^2^ ICB Core Facility, Institute of Biomedical Sciences, University of Sao Paulo, Sao Paulo, SP, 05508-000, Brazil; ^3^ Department of Immunology, Institute of Biomedical Sciences, University of Sao Paulo, Sao Paulo, SP, 05508-000, Brazil; ^4^ Division of Cancer Cell Research, Kanagawa Cancer Center, Yokohama, Kanagawa, 241-8515, Japan; ^5^ Department of Cancer Biology, Vanderbilt University Medical Center, Nashville, TN, 37232, USA; ^6^ School of Dentistry, Positivo University, Curitiba, PR, 81280-330, Brazil

**Keywords:** laminin, invadopodia, β1 integrin, ERK 1-2 pathway, Src kinases

## Abstract

Laminin peptides influence tumor behavior. In this study, we addressed whether laminin peptide C16 (KAFDITYVRLKF, γ1 chain) would increase invadopodia activity of cells from squamous cell carcinoma (CAL27) and fibrosarcoma (HT1080). We found that C16 stimulates invadopodia activity over time in both cell lines. Rhodamine-conjugated C16 decorates the edge of cells, suggesting a possible binding to membrane receptors. Flow cytometry showed that C16 increases activated β1 integrin, and β1 integrin miRNA-mediated depletion diminishes C16-induced invadopodia activity in both cell lines. C16 stimulates Src and ERK 1/2 phosphorylation, and ERK 1/2 inhibition decreases peptide-induced invadopodia activity. C16 also increases cortactin phosphorylation in both cells lines. Based on our findings, we propose that C16 regulates invadopodia activity over time of squamous carcinoma and fibrosarcoma cells, probably through β1 integrin, Src and ERK 1/2 signaling pathways.

## INTRODUCTION

During tumor progression and metastasis, neoplastic cells may detach from primary tumors and migrate into surrounding tissues and blood vessels [1]. These events involve tumor cell interactions with extracellular matrix (ECM) and basement membrane, a specialized structure composed mainly of laminins, type IV collagen, nidogen, and heparan sulfate proteoglycans [2].

Basement membrane degradation is a feature of malignant tumors, and promotes breakdown of mechanical barriers to support cancer cell invasiveness [3, 4]. Matrix metalloproteinases (MMPs) are crucial for basement membrane degradation and tumor dissemination [3, 4]. These enzymes also release protein domains with bioactive activities [5]. Fragments and bioactive peptides can be found in most extracellular matrix proteins [6, 7].

Laminin-111, a basement membrane glycoprotein associated with cell adhesion, migration, and differentiation [8], presents different bioactive sites, which can influence tumor behavior [9–14]. Among these peptides, C16 (short arm of γ1 chain) is involved in migration, angiogenesis, protease activity and metastasis [9, 15, 16].

The initial step of neoplastic cell invasion is characterized by extension of invadopodia, finger-like actin-rich protrusions with intrinsic ECM degradation activity [17–21]. We have previously shown that laminin-derived peptides increase invadopodia activity in a salivary gland adenocarcinoma cell line [22]. However, C16 effects in invadopodia formation and dynamics of other carcinoma types have not been analyzed.

Here we assessed the role of laminin-111-derived peptide C16 in invadopodia activity of cells (CAL27) derived from human oral squamous cell carcinoma. This tumor represents 90% of neoplasms arising from oral epithelium and exhibits a poor prognosis [23, 24]. Moreover, we searched for receptors and signaling pathways related to peptide-induced effects in these cells. For comparative reasons, we also investigated C16-induced effects in a human fibrosarcoma cell line (HT1080). Fibrosarcoma is characterized by proliferation of immature fibroblasts and is well known for its aggressive phenotype, exuberant invasion and increased protease secretion [25–28].

## RESULTS

### C16 stimulates invadopodia activity in tumor cells

**Figure 1:**
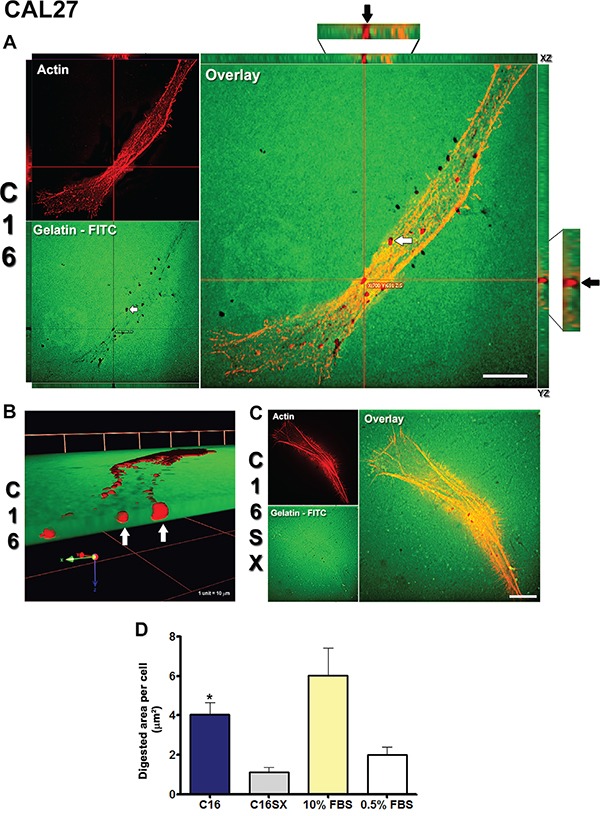
C16 increases invadopodia activity in CAL27 cells In fluorescent substrate degradation assay, cell staining with phalloidin identifies actin filaments (A, C - actin panel). Digestion activity is shown as dark areas on gelatin-FITC background (**A.**, white arrow in gelatin-FITC panel). Red lines (A, overlay panel) indicate points of XY image projected to generate orthogonal planes XZ and YZ. Colocalization of actin protrusions with digested areas (invadopodia) is evident in overlay panel (A, white arrow) and in orthogonal projections XZ and YZ (A, black arrows in orthogonal projections magnifications). Three-dimensional reconstructions of C16-treated sample exhibit actin projections invading gelatin-FITC substrate (**B.**, white arrows). CAL27 cells treated by scrambled control peptide C16SX exhibit few digested foci **C.** Measurements of digested areas per cell demonstrate that invadopodia activity induced by C16 is increased compared to C16SX **D.** Results represent mean ± standard error of 20 cells, in three different experiments. Scale bars: 10 μm.

**Figure 2:**
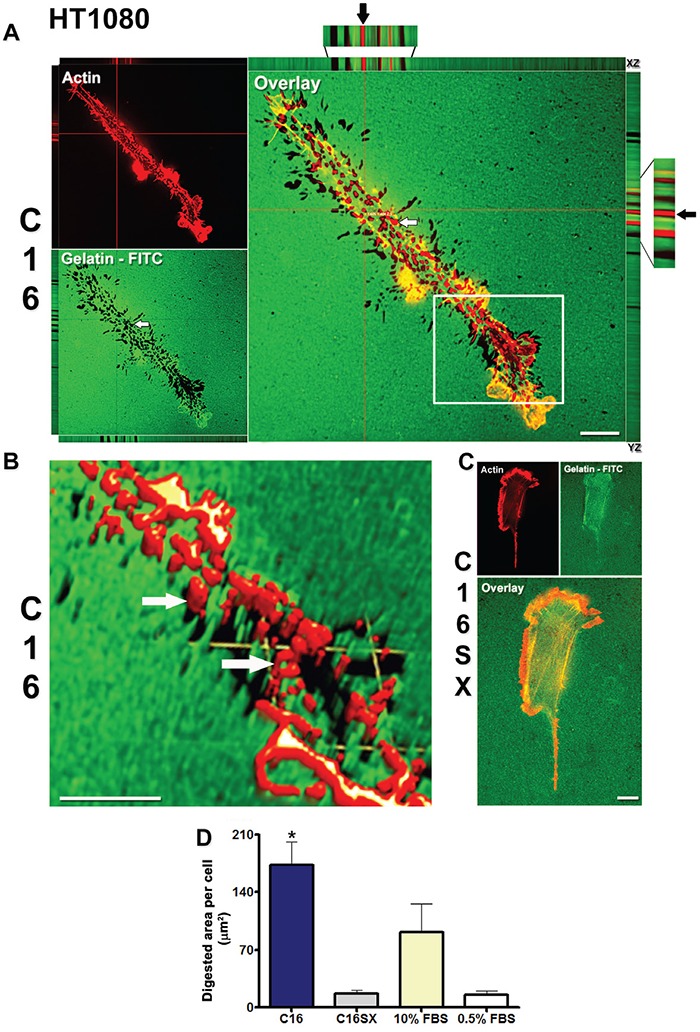
C16 enhances invadopodia activity in HT1080 cells Digestion activity is more prominent in cells treated by C16 (**A.**, white arrow in gelatin-FITC panel). Red lines (A, overlay panel) indicate points of XY image projected to generate orthogonal planes XZ and YZ. Colocalization of actin protrusions with digested areas (invadopodia) is evident in overlay panel (A, white arrow), and in orthogonal projections XZ and YZ (A, black arrows in orthogonal projections magnifications). Figure B is a three-dimensional reconstruction from the boxed area in A, overlay panel. C16-treated sample exhibits actin projections invading the gelatin-FITC substrate (**B.**, white arrows). HT1080 cells treated by C16SX control peptide exhibit few areas of degraded substrate **C.** Measurements of digested areas per cell demonstrate that invadopodia activity induced by C16 is 11-fold increased compared to C16SX **D.** Results represent mean ± standard error of 20 cells, in three different experiments. Scale bars: 10 μm.

A fluorescent ECM substrate degradation assay showed that C16 increased invadopodia activity in both CAL27 (Figure 1A) and HT1080 (Figure 2A) cells. In this assay, invadopodia activity is represented by dark spots (digested areas) in the fluorescent substrate (Figures 1A, 2A - white arrows in gelatin-FITC). In C16-treated samples, we observed prominent invadopodia protrusions, outlined by actin staining (Figures 1A, 2A - actin) colocalized with areas of gelatin digestion (Figures 1A, 2A, white arrows in overlay). Orthogonal projections (Figures 1A, 2A - XZ and YZ) show actin cores penetrating digested spots in the gelatin matrix (Figures 1A, 2A - black arrows). Three-dimensional reconstructions and volume rendering of C16-treated cells further illustrate that actin projections are protruded into the gelatin-FITC substrate (Figures 1B, 2B - white arrows). In contrast, cells treated with the scrambled peptide control (C16SX) showed negligible invadopodia activity (Figures 1C, 2C). Measurements of digested areas per cell demonstrated that C16-treated CAL27 cells had a 3-fold increase in degradation activity compared to CAL27 cells treated with C16SX scrambled control peptide (Figure 1D). Similarly, HT1080 cells treated with C16 peptide exhibited a 11-fold increment in invadopodia activity (Figure 2D). Fibrosarcoma cells presented a more pronounced gelatin digestion activity, being 45 times higher compared to CAL27 cells. C16-induced invadopodia activity was also increased compared to non-peptide negative controls (0.5% FBS) in both cell lines, and to positive control (10% FBS) in HT1080 cells (Figures 1D, 2D). Serum contains growth factors that stimulate invasion and invadopodia, thus it was included as control [53, 54].

### C16 enhances invadopodia activity over time

**Figure 3:**
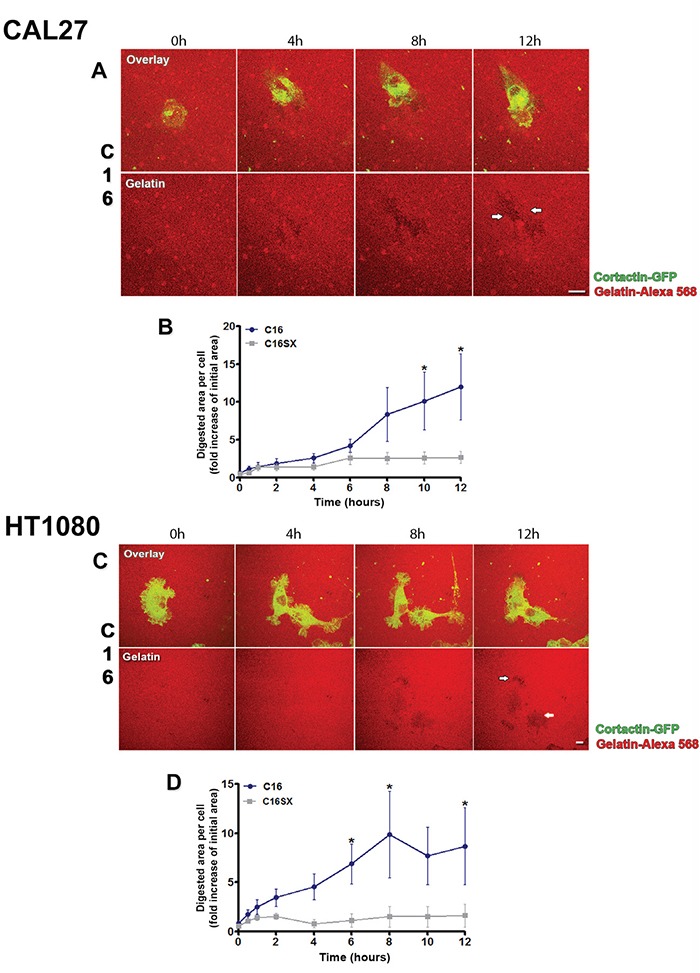
C16 regulates invadopodia activity over time in tumor cells Time-lapse image sequence of cells transfected with cortactin-GFP and cultured on Alexa-568 fluorescent gelatin reveal that C16 enhances invadopodia activity in both CAL27 **A.** and HT1080 **C.** cells over time. Evident digestion area in gelatin matrix is observed 12 hours after treatment with the peptide in both cells (A and C, white arrows). Measurements of digested areas per cell (expressed as fold increase of initial digested area) demonstrate that invadopodia activity of C16-treated CAL27 cells is evident 6 hours after C16 treatment **B.**, while in HT1080 cells, this activity increases 2 hours after peptide treatment **D.** Asterisks in B and D indicate significant data compared to controls (P<0.05). Results represent mean ± standard error of 10 cells. Scale bars: 10 μm.

Invadopodia activity over time in tumor cells treated by C16 was assessed through time-lapse confocal fluorescence microscopy. Time-lapse videos of cortactin-GFP-transfected living cells cultured on Alexa-568 fluorescent gelatin revealed that C16 increases invadopodia activity in both CAL27 (Figure 3A) and HT1080 cells (Figure 3C). In CAL27 cells, measurements of digested spots per cell demonstrated that degradation areas became evident 6 hours after C16 treatment (Figure 3B). After 12 hours, invadopodia activity induced by the peptide was ten times higher than the initial degradation spots (Figure 3B and Movie 1 in Supplemental Material). C16 scrambled control peptide (C16SX) failed to increase invadopodia activity over time (see Movie 1 in Supplemental Material).

C16-treated HT1080 cells exhibited an earlier increase in digested foci, 2 hours after peptide treatment (Figure 3D). After 12 hours, C16 augmented invadopodia activity by 8-fold (Figure 3D and Movie 2 in Supplemental Material). C16 scrambled control peptide (C16SX) failed to increase invadopodia activity over time (see Movie 2 in Supplemental Material).

### β1 integrin may be related to invadopodia activity induced by C16 in tumor cells

**Figure 4:**
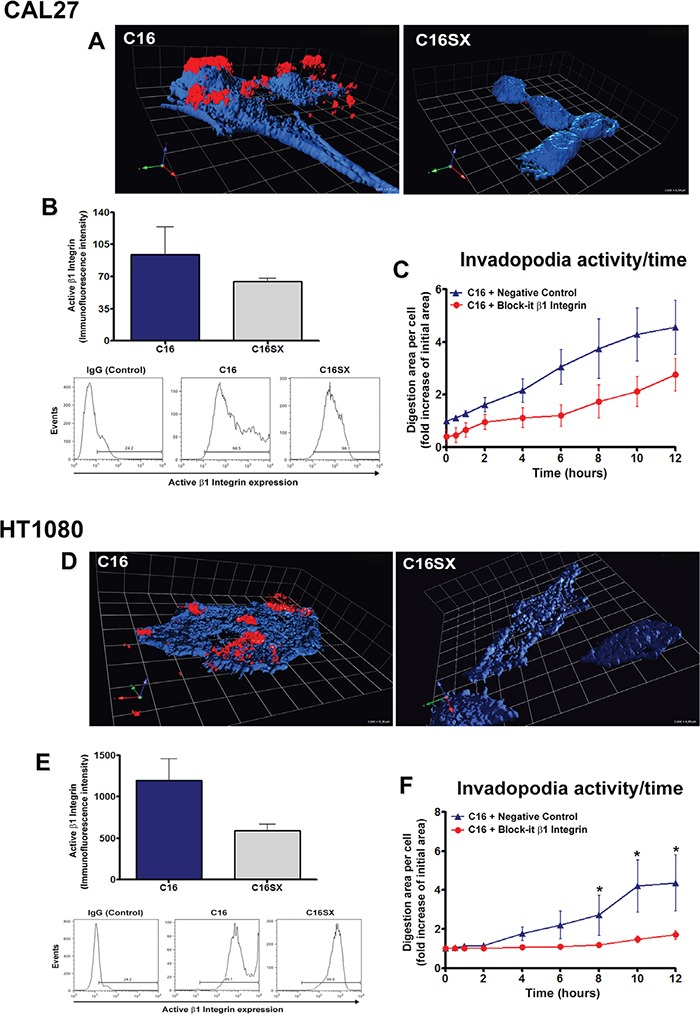
β1 integrin regulates invadopodia activity induced by C16 in tumor cells In 3D reconstructions of fluorescence assays with rhodamine-conjugated peptides (**A** and **D.**, C16 and C16SX panels), cell staining with phalloidin (blue channel) identifies actin filaments. C16 (red channel) is found decorating the cell edge (A and D, C16), while C16SX apparently do not adhere to cell surface (A and D, C16SX). Active β1 integrin expression is increased in both CAL27 and HT1080 C16-treated cells compared to C16SX, as demonstrated by flow cytometry (**B** and **E.**). Tumor cells were transfected with a plasmid containing β1 integrin miRNA (Block-iT, Invitrogen) and subjected to fluorescent substrate degradation assays (**C** and **F.**). Measurements of digested areas per cell show a decrease in invadopodia activity over time in β1 integrin-silenced cells treated with C16, compared to negative control (pcDNA 6.2-GW/EmGFPmiR-neg control – Block-iT). Results in B and E represent mean ± standard error of three experiments. Results in C and F represent mean ± standard error of 10 cells.

In order to better understand how C16 interacts with tumor cells, we carried out fluorescence assays using rhodamine-conjugated peptides and actin-labeled cells. Through confocal fluorescence microscopy and 3D reconstruction samples, we observed that C16 decorates the cell membrane of CAL27 (Figure 4A) and HT1080 cells (Figure 4D). On the other hand, rhodamine-conjugated C16SX was not found at cell edges in either cell line (Figures 4A and 4D, C16SX panel).

Since C16 was observed mainly over the cell surface rather than being endocytosed, we may infer that this peptide would interact with cell membrane receptors. β1 integrin has been suggested as a potential C16 ligand [15]. Therefore, we decided to investigate the role of this integrin subunit in C16-induced invadopodia activity. Initially we analyzed whether this peptide would stimulate β1 integrin activation, using monoclonal antibody (12G10 clone, Santa Cruz Biotechnology). This antibody recognizes an epitope exposed when the integrin is opened or activated upon ligand binding [29]. Flow cytometry demonstrated that C16 increases β1 integrin activation compared to C16SX control peptide in both CAL27 (Figure 4B) and HT1080 cells (Figure 4E).

We also analyzed invadopodia activity through time-lapse microscopy, using tumor cells transfected with GFP-coding plasmids containing a miRNA sequence that promotes β1 integrin knockdown (Block-iT Pol II miR RNAi Expression Vector with EmGFP, Invitrogen). Our data demonstrated that in C16-treated samples, β1 integrin silencing decreased invadopodia activity over time compared to cells transfected with a negative control plasmid in both CAL27 (Figure 4C and Movie 3 in Supplemental Material) and HT1080 cells (Figure 4F).

### Src and ERK 1/2 signaling pathways may be related to C16-induced invadopodia activity in tumor cells

**Figure 5:**
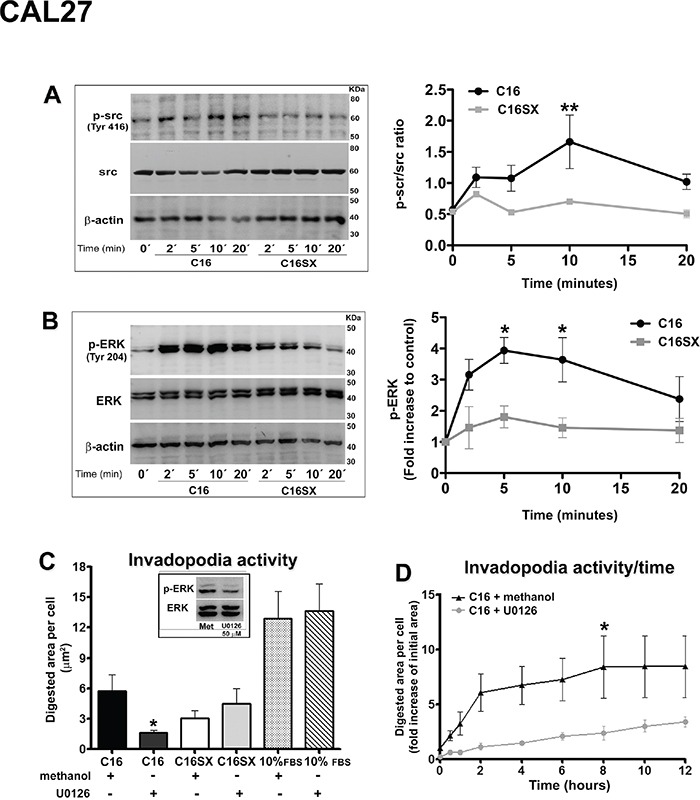
Src and ERK signaling pathways may be involved in C16-induced effects in CAL27 cells Immunoblot and densitometry show that C16 stimulates phosphorylation of Src **A.** and ERK **B.** in CAL27 cells, compared to C16SX-treated samples. A decrease in C16-mediated invadopodia formation is observed in CAL27 cells with ERK 1/2 signaling pathway inhibited by U0126 **C.** This reduction in invadopodia activity is also observed over time, in cortactin-GFP-transfected CAL27 cells treated with U0126 and incubated with C16 **D.** No significant differences are detected in ERK-inhibited cells incubated with C16SX and positive non-peptide control (C). Immunoblot confirms U0126 inhibition efficiency (C, box). Asterisks indicate significant data compared to controls (*P<0.05, **P<0.01). Results in A and B represent mean ± standard error of three different experiments. Results in C represent mean ± standard error of 20 cells, in three different experiments. Results in D represent mean ± standard error of 10 cells.

**Figure 6:**
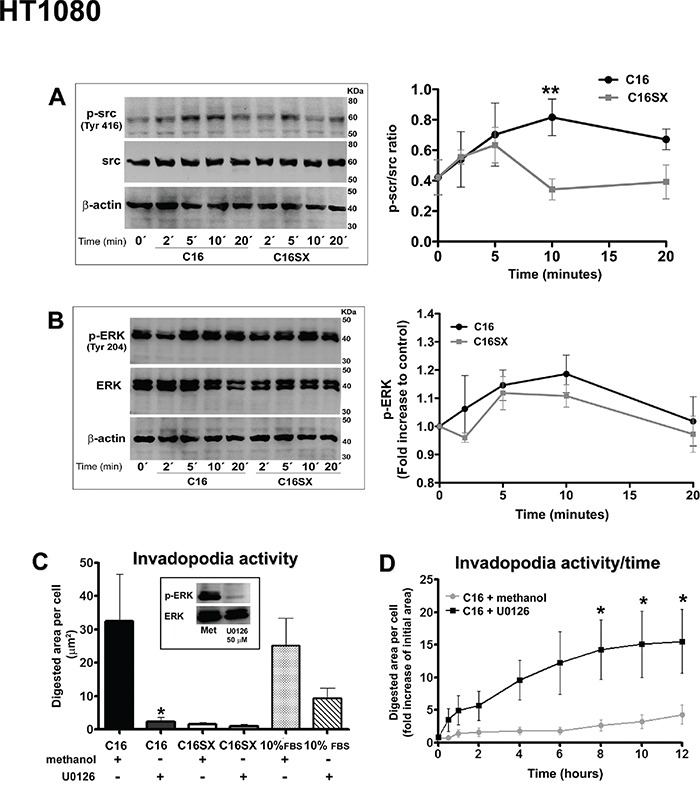
Src and ERK signaling pathways may be involved in C16-induced effects in HT1080 cells Immunoblot and densitometry show that C16 stimulates Src phosphorylation in HT1080 cells **A.** Increased ERK phosphorylation is consistently observed in immunoblot, but not significant in densitometry **B.** A decrease in C16-mediated invadopodia formation is observed in HT1080 cells with ERK 1/2 signaling pathway inhibited by U0126 **C.** This reduction in invadopodia activity is also observed over time, in cortactin-GFP-transfected HT1080 cells treated with U0126 and incubated with C16 **D.** No differences are detected in ERK-inhibited cells incubated with C16SX and positive non-peptide control (C). Immunoblot confirms U0126 inhibition efficiency (C, box). Asterisks in C, D indicates significant data compared to methanol (vehicle) controls (P<0.05). Results in A and B represent mean ± standard error of three different experiments. Results in C represent mean ± standard error of 20 cells, in three different experiments. Results in D represent mean ± standard error of 10 cells.

We investigated signaling pathways related to C16. It has been shown that Src signaling cascade is essential for invadopodia assembly [30]. C16 increased Src phosphorylation in both Cal27 (Figure 5A) and HT1080 cells (Figure 6A). Src phosphorylation increase was significant 10 minutes after peptide addition.

Src phosphorylation may induce the activation of ERK pathway [31]. Furthermore, ERK kinase activation can be associated with the effects of some laminin peptides, such as MMP secretion and invadopodia activity [13, 22]. C16 significantly increased ERK 1/2 Tyr-204 phosphorylation 5 and 10 minutes after peptide addition. (Figure 5B). Increased ERK phosphorylation in HT1080 cells was consistently observed in immunoblot, but not significant in densitometry. Moreover, a decrease in C16-mediated invadopodia activity was observed in CAL27 (Figure 5C) and HT1080 cells (Figure 5C) treated with MEK inhibitor U0126, compared to cells treated with the inhibitor vehicle (methanol) and treated with C16. No significant differences were detected in ERK-inhibited cells incubated with scrambled peptide C16SX (Figure 5C and 6C). MEK blocker does not inhibit invadopodia activity in cells treated with 10% FBS. We may suggest that FBS and C16 would trigger different signaling mechanisms. Immunoblot confirmed inhibition of ERK phosphorylation (Figures 5C and 6C, box).

The effect of ERK inhibition in invadopodia activity was also observed in cortactin-transfected tumor cells through time-lapse experiments. In C16-treated CAL27 samples, cells incubated with U0126 decreased invadopodia activity over time, compared to controls (see Movie 4 in Supplemental Material). A similar result was observed in HT1080 cells (see Movie 5 in Supplemental Material). Measurements of digested area per cell demonstrated that ERK inhibition in cells treated with C16 decreased invadopodia activity of tumor cells over time (Figure 5D and 6D).

### C16 promotes cortactin phosphorylation in tumor cells

**Figure 7:**
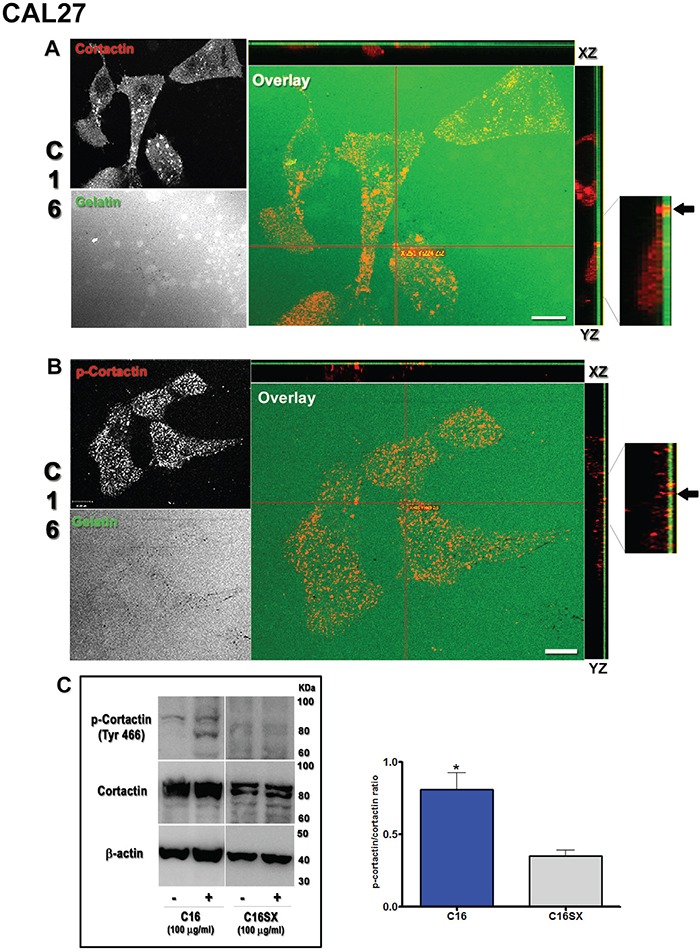
C16 stimulates cortactin phosphorylation in CAL27 cells In C16-treated samples, cortactin (red channel) is observed as a diffuse cytoplasmic staining (**A.**, orthogonal projections XZ and YZ in overlay panel) with sporadic accumulations. Phospho-cortactin (red channel) is located mostly on the ventral membrane of cells (**B.**, orthogonal projections XZ and YZ in overlay panel). Cortactin and phospho-cortactin are found in digested areas (A and B, overlay panel and black arrows in YZ magnifications). Red lines in A and B indicate points of XY image projected to generate orthogonal planes XZ and YZ. Immunoblot **C.** shows that C16 stimulates cortactin phosphorylation compared to C16SX. Asterisks indicate significant data compared to controls (P<0.05). Results in C represent mean ± standard error of three different experiments. Scale bars: 10 μm.

Src kinases activate invadopodia key molecules, like cortactin. Phosphorylation status of cortactin controls invadopodia formation and assembly [32, 33]. Therefore, we also investigated whether peptide C16 would influence distribution and phosphorylation of cortactin in tumor cells. Immunofluorescence demonstrated that, in CAL27 cells treated with C16 (Figure 7A), cortactin was diffusely distributed throughout the cytoplasm. Phospho-cortactin (Tyr-466), on the other hand, presented a trend to concentrate on ventral portions of tumor cells (Figure 7B, orthogonal projections XZ and YZ). Colocalization of cortactin and phospo-cortactin was observed in gelatin digested foci (Figure 7, black arrows in YZ orthogonal projection magnifications). We found similar results for HT1080 cells (see Supplemental Figure 1).

Immunoblot revealed that C16 stimulates cortactin phosphorylation in tumor cells. CAL27 samples showed a prominent level of cortactin phosphorylation in the presence of C16, compared to control C16SX-treated samples (Figure 7C - C16). Densitometry showed a significant increase in phospho-cortactin/cortactin ratio in samples treated with C16 (Figure 7C – graph). Total cortactin levels were increased in comparison to C16SX-incubated samples in both cell lines. Similar results were also observed in HT1080 cells (see Supplemental Figure 1).

## DISCUSSION

We showed that laminin-111 peptide C16 increased invadopodia activity over time and stimulated cortactin phosphorylation in cell lines derived from squamous cell carcinoma (CAL27) and fibrosarcoma (HT1080), two invasive tumors with poor prognosis [23, 27]. We searched for regulatory mechanisms underlying C16-mediated effects in CAL27 and HT1080 cells. A series of experiments suggested that β1 integrin subunit, and Src and ERK signaling cascades regulate C16-related invadopodia formation and/or activity in both cells. We have previously shown that C16 increased invadopodia in a salivary adenocarcinoma cell line [22]. However, to our knowledge, this is the first report addressing the effect of a laminin peptide in invadopodia formation and matrix degradation rate over time. Furthermore, the role of C16 increasing both Src and cortactin phosphorylation are also novel insights on invadopodia activity.

During tumor dissemination, ECM molecules modulate cancer cells behavior [34]. Laminins, heterotrimeric ECM proteins composed of α, β and γ chains, influence tumor invasiveness [35, 36]. Laminins may undergo controlled proteolysis during tumor-induced basement membrane breakdown, releasing peptides with biological activities [5, 7]. Laminin fragments have been detected in serological exams of head and neck squamous cell carcinoma patients [37], corroborating the hypothesis that tumor-mediated enzymatic degradation can promote laminin peptides release.

Different active sequences on laminin-111 have been described [38, 39]. C16, located on the short arm of γ1 chain, is involved in cell adhesion and differentiation [16, 40], angiogenesis [15, 41], migration, MMP secretion, and metastasis of melanoma cells [9]. Laminin isoforms containing γ1 chain, where C16 peptide is located, are found in oral epithelium and blood vessel basement membranes [42, 43]. Therefore, basement membrane proteolysis, both in initial invasion steps and in tumor cell intravasion to blood vessels may induce laminin γ1 cleavage and C16 release. In addition, mass spectrometry studies using MCF10A cells demonstrated that an amino acid sequence similar to C16 (except for the absence of the two last amino acids in the carboxy-terminal end) was generated by MMP9-mediated laminin-111 cleavage, thus indicating that this peptide is a product of cell-induced proteolysis [44].

Invadopodia are required in almost all stages of tumor invasion and metastatic dissemination [45]. Invadopodia are typical protrusive structures that may be found in cancer cells, and are composed of an actin-rich core surrounded by adhesion and scaffolding proteins [18, 46]. Using invadopodia, cells can synchronize ECM degradation and cell motility, allowing cell migration through the tissues [19].

Invadopodia have been identified in tumor cell lines from melanoma, breast carcinoma, glioma and head and neck squamous cell carcinoma [25, 47, 48]. Through fluorescent matrix degradation assay we verified that C16 increased invadopodia activity in CAL27 and HT1080 cells. Fibrosarcoma cells presented a higher invadopodia activity rate, probably due to pronounced aggressive characteristics and elevated MMP secretion related to this tumor [26].

To clarify how invadopodia formation in tumor cell lines evolves during C16 treatment, we utilized time-lapse confocal imaging using GFP-cortactin-transfected cells cultured on fluorescent gelatin. In both cell lines C16 increased invadopodia activity over time compared to controls. Measurement of digested areas over time showed that the peptide starts to induce matrix degradation after 2 hours in HT1080 cells, and after 6 hours in CAL27 cells. This finding may explain the prominent differences observed between invadopodia-related digested areas of these two cell lines.

To better evaluate how C16 would interact with tumor cells we assessed its cellular location using rhodamine-conjugated peptides. We observed that C16 decorates the edges of tumor cells, suggesting that this peptide may adhere to cell receptors. It is well known that integrins mediate laminin effects in cell behavior [49]. β1 integrin was previously suggested as putative C16 ligand [15]. It has been recently found that β1 integrin promotes exocytosis of MT1-MMP at invadopodia, thereby increasing invadopodia activity [46]. Using FACS analysis with an antibody that recognizes active β1 integrin, we demonstrated that C16 induces β1 integrin activation in CAL27 and HT1080 cells. Moreover, β1 integrin inhibition in both cell lines decreased C16-related invadopodia activity over time. Therefore, we may infer that this integrin subunit regulates invadopodia formation.

Signals generated by C16 may be transduced by integrins and activate different signaling pathways [19]. We observed that C16 stimulates Src phosphorylation in both CAL27 and HT1080 cells just few minutes after treatment. Furthermore, Src phosphorylation may activate ERK pathway [31]. We previously demonstrated that ERK increases invasion, MMPs secretion and invadopodia activity induced by laminin peptides [13, 22]. Our results showed that C16 increased ERK phosphorylation, corroborating a possible relationship between Src and MAP kinases signaling pathways. A detailed study of this molecular machinery involving cell membrane receptors and signaling pathways will be the target of our future investigations.

Src kinases are responsible for the activation of some invadopodia key molecules, like cortactin [47]. It is known that the phosphorylation status of cortactin control invadopodia formation and assembly [32, 33]. C16 increased cortactin phosphorylation (Tyr 466) in both CAL27 and HT1080 cells. We also observed that phospho-cortactin tends to localize in the cell-gelatin interface in C16-treated samples.

**Figure 8:**
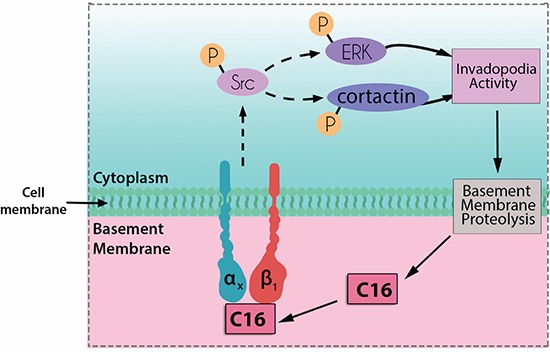
Diagram showing cellular events related to invadopodia formation stimulated by laminin peptide C16 Based on our results, we may infer that C16 may interact with integrin dimers containing the β1 subunit, prompting to Src phosphorylation. Src activation would mediate ERK and cortactin phosphorylation, which may contribute to invadopodia formation. Invadopodia could induce basement membrane proteolysis, promoting C16 release from the intact laminin molecule, and newly-released peptides would then be available to interact with tumor cells. Dashed lines indicate indirect interactions between molecules.

The biological roles of laminin-derived peptides such as C16 in tumor cell behavior have not been fully elucidated. Here, we tried to connect some biological events, to better understand how this peptide regulates tumor invasiveness. Figure 8 summarizes our working hypothesis regarding C16-induced invadopodia formation in CAL27 and HT1080 cells. Based on our findings, we may infer that, in early invasion steps, tumor cell-associated invadopodia would be responsible for basement membrane breakdown, leading to laminin cleavage. C16 and other laminin bioactive peptides would be released by proteolytic processing and become available to exert their cellular effects. C16 would interact with integrin heterodimers containing the β1 subunit and stimulate Src phosphorylation, directly or through interactions with other molecules. Src would promote the phosphorylation of ERK 1/2 and cortactin, thus inducing new invadopodia formation.

## MATERIALS AND METHODS

### Peptides

EZ Biolab (Westfield, IN, USA) synthesized peptide C16 (KAFDITYVRLKF) and scrambled control peptide C16SX (FKLRVYTIDFAK). Peptides purity was 98% (RP-HPLC), and molecular mass was confirmed by mass spectrometry.

### Cell culture

CAL27 cell line, derived from a human squamous cell carcinoma from the tongue [50], was cultured in Dulbecco's modified Eagle's medium (DMEM, Sigma Chemical Corp., St. Louis, MO, USA) supplemented with 10% fetal bovine serum (FBS, Vitrocell, Campinas, SP, Brazil). HT1080 cell line, from human fibrosarcoma [51], was cultured in minimum Eagle's medium (MEM, Vitrocell) with 10% FBS. Both cell lines were maintained in 75-cm^2^ flasks in a humidified atmosphere of 5% CO_2_ at 37°C.

### Fluorescent substrate degradation assay

To assess the role of laminin-111 peptide C16 in invadopodia activity of CAL27 and HT1080 cells, fluorescent gelatin substrate degradation assays were carried out. Gelatin-FITC substrates (Invitrogen, Eugene, OR, USA) were prepared over glass coverslips, as previously described [52]. Cells (2 × 10^5^) were plated on gelatin substrates and incubated in medium with 0.5% fetal bovine serum (FBS) at 37°C for 1-3 hours. After attachment of cells to substrate, C16 (100 μg/ml) was added to the medium and samples were incubated overnight (16 hours). Control cells were treated with scrambled control peptide C16SX. Non-peptide controls included cells cultured with either 0.5% FBS (negative control) or 10% FBS (positive control). Serum contains growth factors that stimulate invasion and invadopodia, thus it was included as control [53, 54].

Cells were fixed in 4% paraformaldehyde in PBS for 15 minutes and permeabilized using 0.05% Triton X-100 in PBS for 5 minutes. Cells were labeled to actin by the specific probe rhodamine-phalloidin (Invitrogen) and mounted with Pro Long (Invitrogen).

Samples were analyzed by widefield fluorescence microscope equipped with a 100x PlanApo 1.4 NA objective (Axiophot, Carl Zeiss, Oberkochen, Germany), equipped with a digital CCD camera (CoolSnap HQ2, Photometrics Inc, Tucson, AZ, USA). To assess invadopodia extensions through the fluorescent substrate, at least ten Z sections per sample field were obtained using a piezoelectric device (PIFOC, Physik Instrumente, Germany) coupled to the objective. The microscope and all devices were controlled by Metamorph Premier 7.6 software (Molecular Devices, Sunnyvale, CA, USA).

A total of 20 random cells were imaged per experimental group. Invadopodia were characterized as actin protrusions (red channel) superimposed to digested areas (dark spots) in fluorescent gelatin substrate (green channel). Measurement of gelatin digested area per cell was estimated using ImageJ public domain software (https://gdc-portal.nci.nih.gov/). Three-dimensional reconstructions, orthogonal projections and image restoration through deconvolution algorithms were carried out by Volocity software (PerkinElmer, Waltham, MA, USA).

Fluorescent substrate degradation assays were carried out at least three times, with consistent results.

### Time-lapse 4D confocal fluoresce microscopy

CAL27 and HT1080 cells were transfected with 300-500 ng of cortactin-GFP cDNA, using Lipofectamine 2000 (Invitrogen). After 24 hours, transfected cells were plated over Alexa 568-conjugated gelatin substrate (Invitrogen) in 10% FBS medium, and incubated in a humidified atmosphere of 5% CO_2_ at 37°C for 3-6 hours prior to time-lapse experiments. Coverslips with transfected cells and fluorescent gelatin were mounted over microscope slides with ultra-thin adhesive gaskets (Bioscience Tools, San Diego, CA, USA), and maintained in media without phenol red (Sigma) containing 1% Nu-serum (BD Biosciences) and either C16 or C16SX peptides (100 μg/ml). Time-lapse videos of living cells were acquired at 15-minute interval, for at least 12 hours, in a Carl Zeiss LSM 780-NLO confocal microscope (CEFAP-ICB-USP), equipped with a large chamber incubation system including controlled temperature, CO_2_ pressure and humidity. Invadopodia extensions (green) through fluorescent substrate (red) were evaluated with acquisition of at least ten Z sections per time point. A total of 10 random fields were imaged per experimental group. Measurement of degraded spots per cell (μm^2^) over time was performed using ImageJ software.

### Cellular localization of C16

Tumor cells were plated over coverslips for 24 hours. Samples were then incubated with rhodamine-conjugated C16 or C16SX (100 μg/ml) in medium containing 0,5% FBS for 1 hour. Cells were then fixed, permeabilized and labeled for actin (with Alexa-633 phalloidin, Invitrogen). Samples were mounted with ProLong Gold (Invitrogen), and analyzed by confocal fluorescence microscope Carl Zeiss LSM 510 (Department of Cancer Biology, Vanderbilt University Medical Center). Three-dimensional reconstructions were carried out by Volocity software (PerkinElmer).

### Flow cytometry

CAL27 and HT1080 cells (5×10^5^) were grown on 35 mm plates overnight, and incubated with C16 or C16SX (100 μg/ml) for 1 hour. Cells were harvested and labeled with mouse monoclonal β1 integrin primary antibody (12G10 clone, Santa Cruz Biotechnology Inc., CA, USA) followed by Alexa Fluor 488 goat anti-mouse secondary antibody (Invitrogen). The 12G10 recognizes an epitope exposed when the integrin is opened or activated upon ligand binding [29].

Cells were analyzed in a FACSCalibur cytometer (Becton Dickinson, San Jose, CA, USA) with CellQuest software (Becton Dickinson). At least 10,000 gated events were acquired per sample analyzed.

### β1 integrin knockdown

To assess whether β1 integrin is related to C16-induced invadopodia activity, CAL27 and HT1080 cells were gown on 6-well plates and transfected with 500 ng of a GFP-coding plasmid containing a β1 integrin siRNA sequence (Block-iT Pol II miR RNAi Expression Vector with EmGFP, Invitrogen), using Lipofectamine 2000 transfection reagent (Invitrogen). Tumor cells were incubated with a mixture containing the plasmid, transfection reagent and Opti-MEM (Gibco) for 48-72 hours prior time-lapse experiments, in which were plated on Alexa 568-conjugated gelatin and treated with C16 (100 μg/ml), as described previously. Control groups included cells transfected with a control plasmid containing a scrambled siRNA sequence (pcDNA 6.2™-GW/EmGFP-miR, Invitrogen) and incubated with C16.

### Immunoblot

CAL27 and HT1080 cells were grown on 6-well plates and incubated with either C16 or C16SX scrambled control peptide for different time intervals. All samples were subjected to immunoblot, as described elsewhere [14]. The following primary antibodies were used: Src – phospho-Tyr 416 (1:1000, Cell Signaling Technology, Beverly, MA, USA), Src (1:1000, Cell Signaling), cortactin – phospho-Tyr 466 (1:1000, Signalway Antibodies, Maryland, MD, USA), cortactin (1:500, Santa Cruz), ERK – phospho-Tyr 204 (1:250, Santa Cruz), ERK 1/2 (1:500, Santa Cruz), or β-actin (1:2000, Sigma). To assess activation of Src and ERK signaling pathways, cells were serum-starved for 24 hours prior to peptide treatment.

### ERK inhibition

Cells cultured overnight in medium with 10% FBS were serum-starved for 24 hours and then incubated with 50 μM of the MEK1/2 inhibitor U0126 (Cell Signaling Technology, Beverly, MA, USA). As a control, cells were incubated with U0126 vehicle (methanol). Both ERK inhibited and control cells were treated by C16 and subjected to fluorescent gelatin substrate degradation assays. ERK inhibition in C16-treated cells was also observed in living cells transfected with cortactin-GFP, incubated with U0126 and subjected to time-lapse experiments. ERK inhibition efficiency was assessed by immunoblot.

### Statistical analysis

Student's t-test was performed to evaluate differences between two groups. Differences between three or more groups were assessed by analysis of variance (ANOVA), followed by Bonferroni's multiple comparisons test. The software used was GraphPad Prism (GraphPad Software, Inc., San Diego, CA).

## SUPPLEMENTARY FIGURE AND MOVIES












