# Supplementary material for: Laminin-111 peptide C16 regulates invadopodia activity of malignant cells through β1 integrin, Src and ERK 1/2
Source: Oncotarget. 2016 Jun 15;7(30):47904–17. doi: 10.18632/oncotarget.10062 (PMC5216987; doi:10.18632/oncotarget.10062)
Supplement: Supplementary file 1 [file oncotarget-07-47904-s001.pdf]

Laminin-111 peptide C16 regulates invadopodia activity of malignant cells through β1 integrin, Src and ERK 1/2

SUPPLEMENTARY MATERIALS

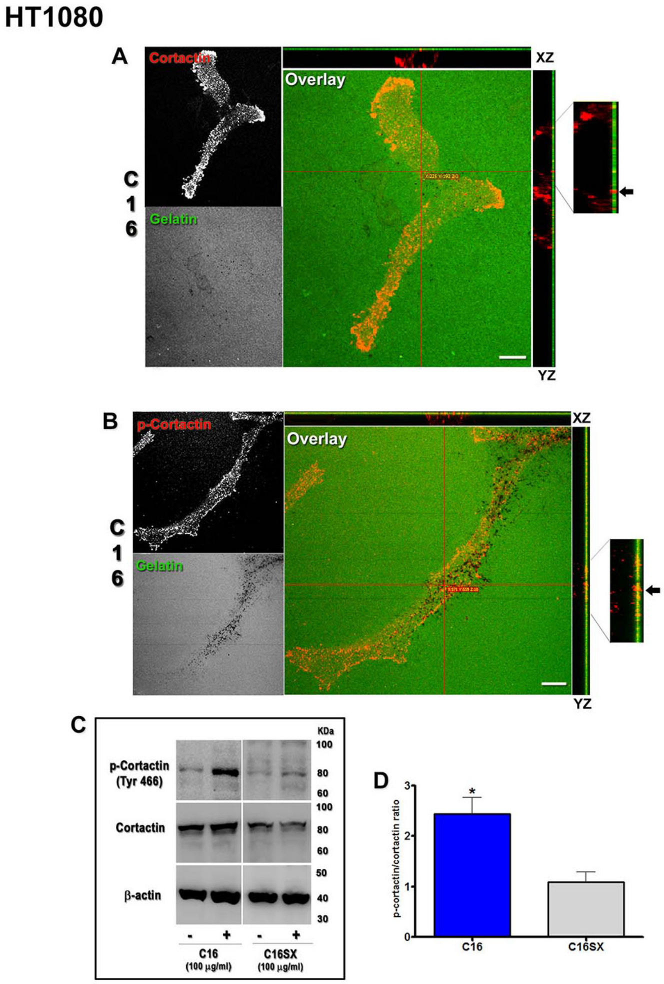

Supplementary Figure S1: C16 stimulates cortactin phosphorylation in HT1080 cells. In C16-treated samples, cortactin (red channel) is observed as a diffuse cytoplasmic staining (**A.**, orthogonal projections XZ and YZ in overlay panel) with sporadic accumulations. Phospho-cortactin (red channel) is located mostly on the ventral membrane of cells (**B.**, orthogonal projections XZ and YZ in overlay panel). Cortactin and phospho-cortactin are found in digested areas (A and B, overlay panel and black arrows in YZ magnifications). Red lines in A and B indicate points of XY image projected to generate orthogonal planes XZ and YZ. Immunoblot **C.** and **D.** shows that C16 stimulates cortactin phosphorylation compared to C16SX. Asterisks indicate significant data compared to controls (P<0.05). Results in C represent mean ± standard error of three different experiments. Scale bars: 10 µm.

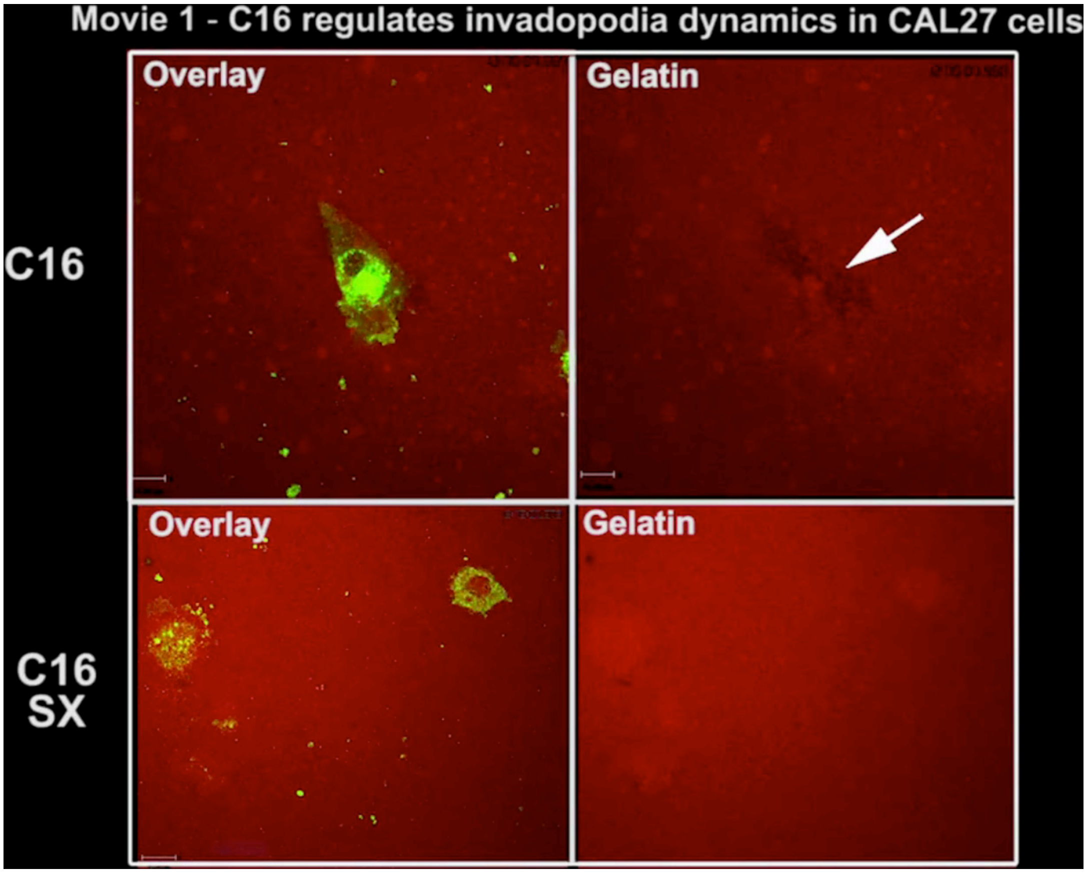

Supplemental Movie 1: C16 regulates invadopodia activity over time in CAL27 cells. Time-lapse video of cells transfected with cortactin-GFP (green channel in Overlay video) and cultured on fluorescent gelatin shows that C16 stimulates substrate digestion over time (top). In the top gelatin video, arrow indicates dark digestion spots. C16SX control peptide fails to stimulate substrate digestion by CAL27 cells (bottom).

See Supplementary File 1

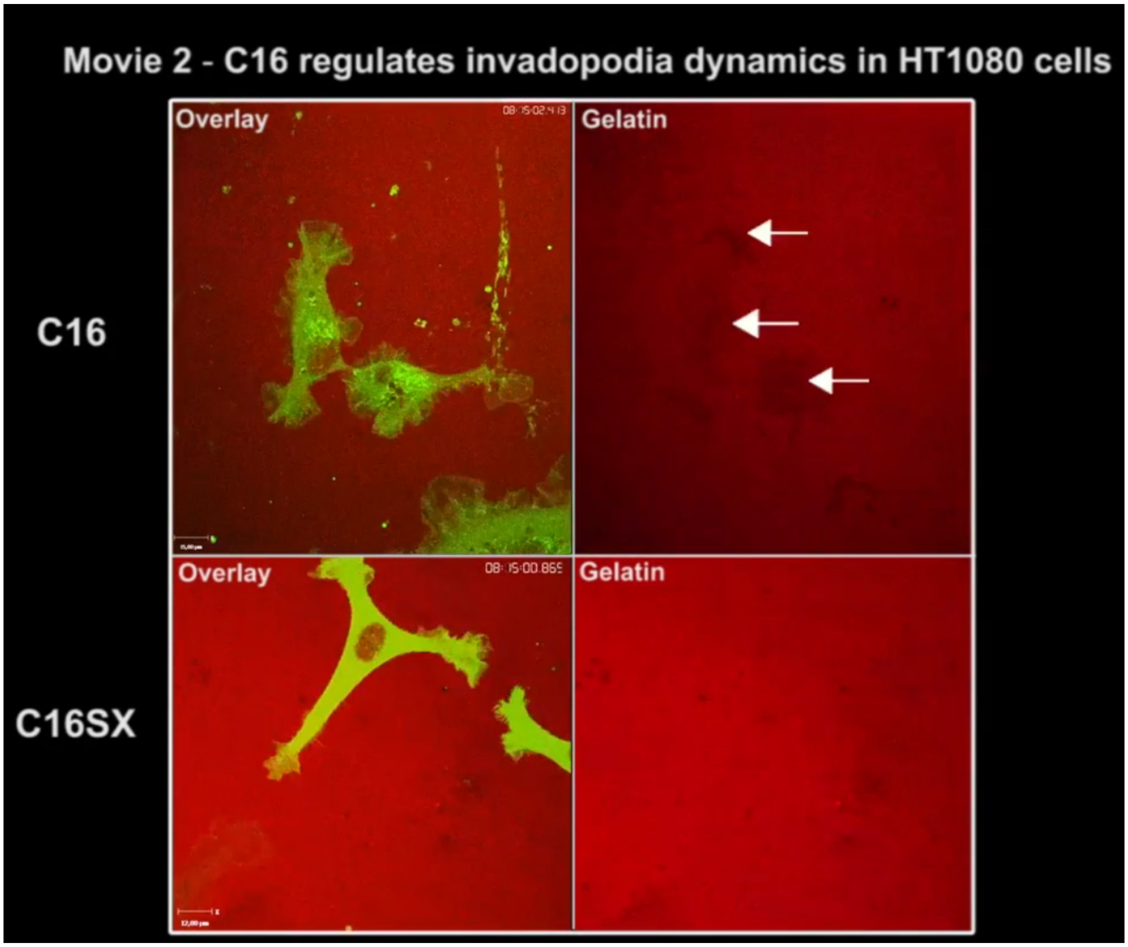

Supplemental Movie 2: C16 regulates invadopodia activity over time in HT1080 cells. Time-lapse video of cells transfected with cortactin-GFP (green channel in Overlay video) and cultured on fluorescent gelatin shows that C16 stimulates substrate digestion over time (top). In the top gelatin video, arrows indicate dark digestion spots. C16SX control peptide fails to stimulate substrate digestion by HT1080 cells (bottom).

See Supplementary File 2

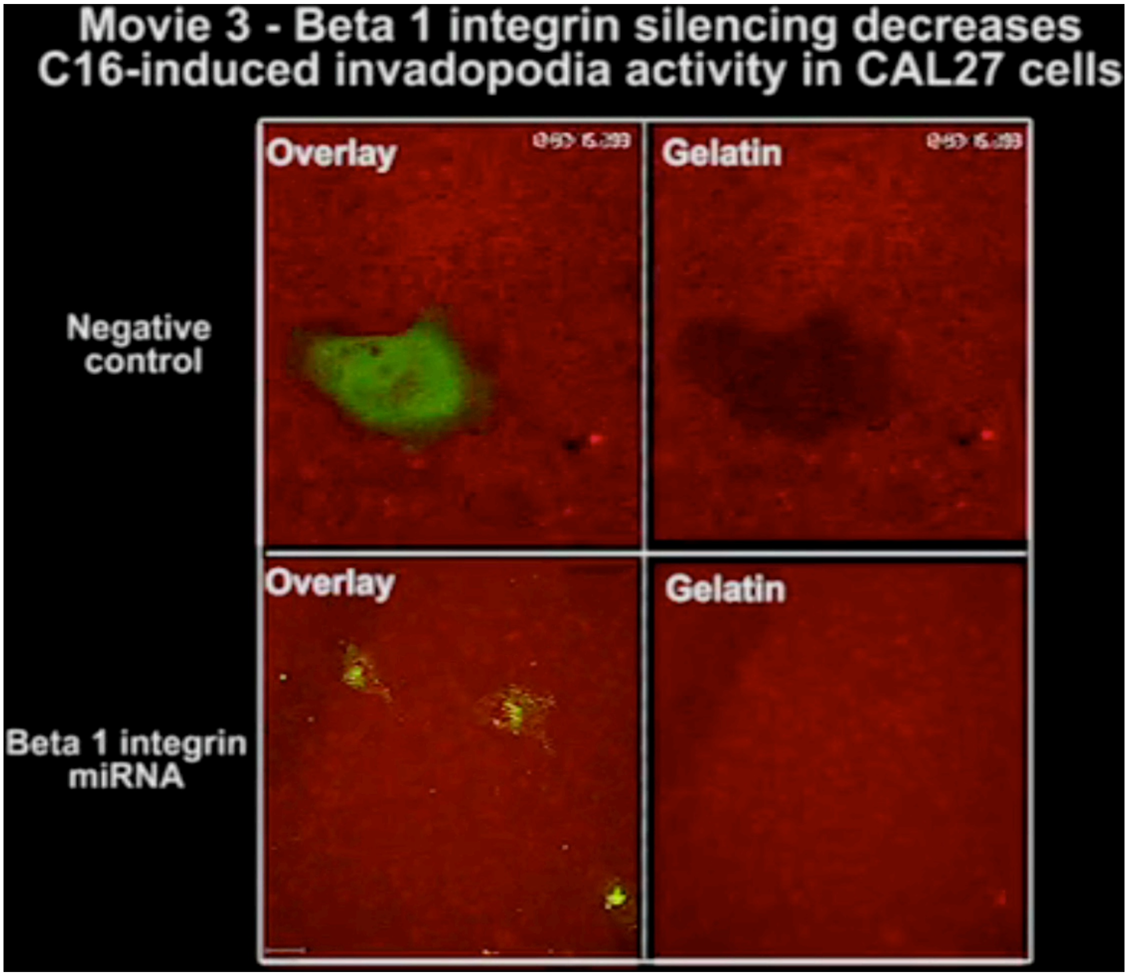

Supplemental Movie 3: β1 integrin silencing decreases C16-induced invadopodia activity over time in CAL27 cells. Time-lapse videos of cells transfected with plasmid containing control miRNA (green in top Overlay video), following treatment by C16. In this situation, C16 maintains its ability to increase invadopodia activity over time (top panels). Cells were also transfected with plasmid containing β1 integrin miRNA (Block-iT, Invitrogen - green in bottom Overlay video). β1 depletion reduced C16-induced invadopodia activity.

See Supplementary File 3

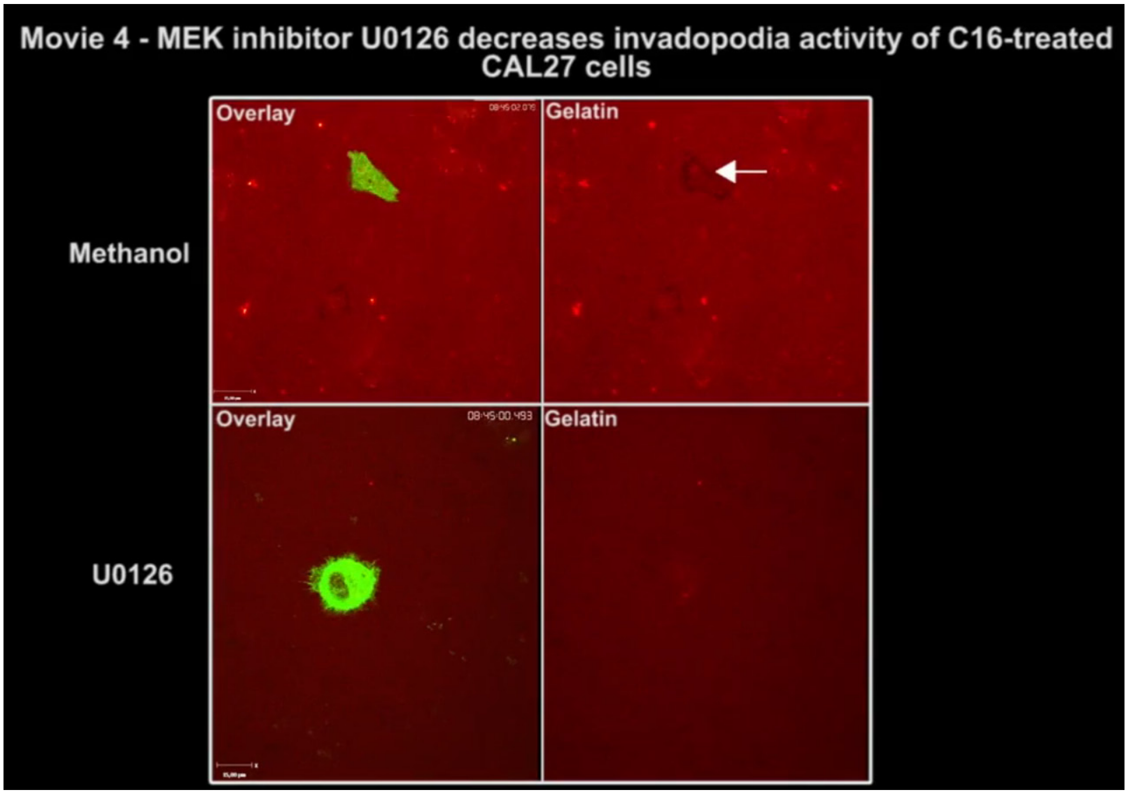

Supplemental Movie 4: MEK inhibitor U0126 decreases invadopodia activity of C16-treated CAL27 cells. Time-lapse video of cortactin-GFP-transfected cell incubated with C16 and methanol (vehicle – negative control) shows increase of C16-induced invadopodia activity over time (top, arrow). Inhibition of ERK signaling pathway by U0126 reduces C16-dependent invadopodia activity (bottom).

See Supplementary File 4

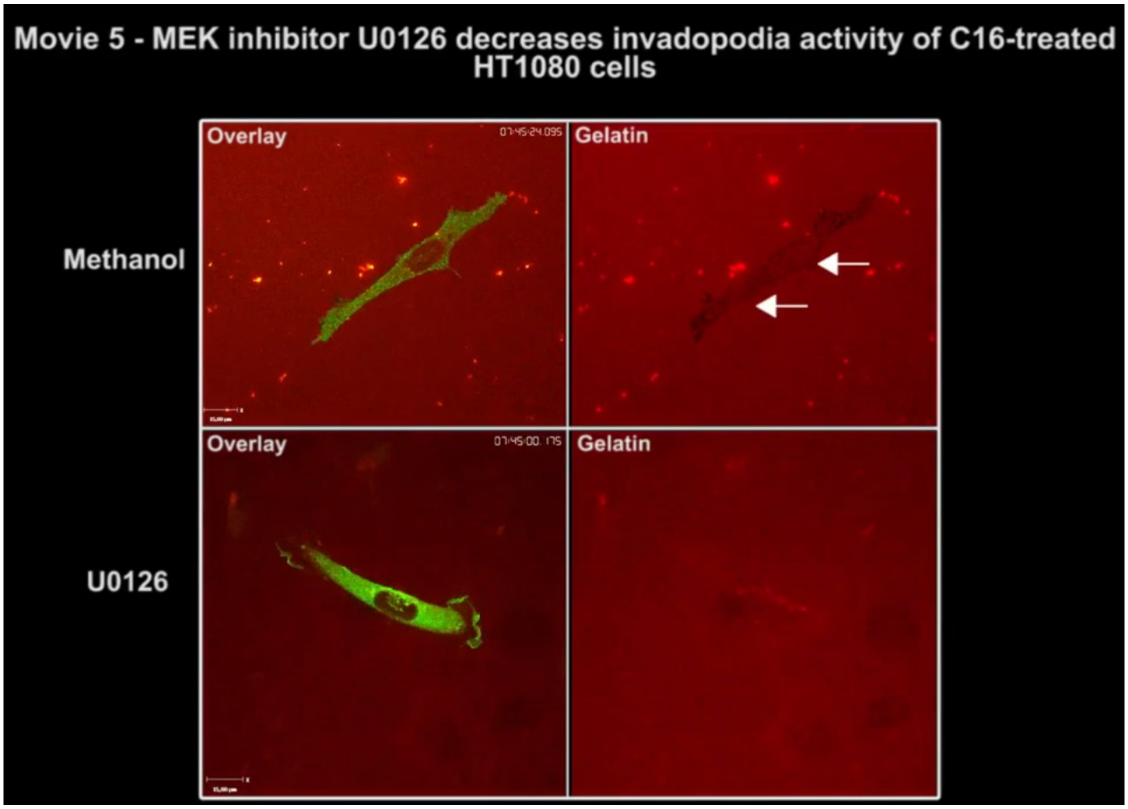

Supplemental Movie 5: MEK inhibitor U0126 decreases invadopodia activity of C16-treated HT1080 cells. Time-lapse video of cortactin-GFP-transfected cell incubated with C16 and methanol (vehicle – negative control) shows increase of C16-induced invadopodia activity over time (top, arrows). Inhibition of ERK signaling pathway by U0126 reduces C16-dependent invadopodia activity (bottom).

See Supplementary File 5
